# Methyl 4-(3-chloro­prop­oxy)-5-meth­oxy-2-nitro­benzoate

**DOI:** 10.1107/S1600536809009076

**Published:** 2009-03-19

**Authors:** Min Zhang, Ran-zhe Lu, Lu-na Han, Wen-bin Wei, Hai-bo Wang

**Affiliations:** a College of Light Industry and Food Science, Nanjing University of Technology, Xinmofan Road No. 5 Nanjing, Nanjing 210009, People’s Republic of China; b College of Science, and College of Light Industry and Food Science, Nanjing University of Technology, Xinmofan Road No. 5 Nanjing, Nanjing 210009, People’s Republic of China

## Abstract

The asymmetric unit of the title compound, C_12_H_14_ClNO_6_, contains two crystallographically independent mol­ecules, in which the benzene rings are oriented at a dihedral angle of 9.12 (3)°. In the crystal structure, weak inter­molecular C—H⋯O hydrogen bonds link the mol­ecules into a three-dimensional network.

## Related literature

For general background, see: Knesl *et al.* (2006 ▶). For bond-length data, see: Allen *et al.* (1987 ▶).
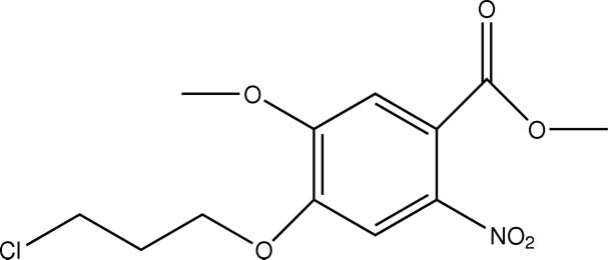


## Experimental

### 

#### Crystal data

C_12_H_14_ClNO_6_
*M* *_r_* = 303.69
Monoclinic, 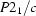

*a* = 23.150 (5) Å
*b* = 15.013 (3) Å
*c* = 8.0700 (16) Å
β = 93.42 (3)°
*V* = 2799.7 (10) Å^3^
*Z* = 8
Mo *K*α radiation
μ = 0.30 mm^−1^
*T* = 294 K
0.30 × 0.20 × 0.20 mm

#### Data collection

Enraf–Nonius CAD-4 diffractometer
Absorption correction: ψ scan (North *et al.*, 1968 ▶) *T*_min_ = 0.916, *T*_max_ = 0.943
5208 measured reflections
5096 independent reflections
2874 reflections with *I* > 2σ(*I*)
*R*_int_ = 0.038
3 standard reflections frequency: 120 min intensity decay: 1%

#### Refinement

*R*[*F* ^2^ > 2σ(*F* ^2^)] = 0.065
*wR*(*F* ^2^) = 0.162
*S* = 1.03
5096 reflections
362 parameters
H-atom parameters constrained
Δρ_max_ = 0.40 e Å^−3^
Δρ_min_ = −0.30 e Å^−3^

### 

Data collection: *CAD-4 Software* (Enraf–Nonius, 1989 ▶); cell refinement: *CAD-4 Software*; data reduction: *XCAD4* (Harms & Wocadlo, 1995 ▶); program(s) used to solve structure: *SHELXS97* (Sheldrick, 2008 ▶); program(s) used to refine structure: *SHELXL97* (Sheldrick, 2008 ▶); molecular graphics: *ORTEP-3 for Windows* (Farrugia, 1997 ▶); software used to prepare material for publication: *SHELXTL* (Sheldrick, 2008 ▶).

## Supplementary Material

Crystal structure: contains datablocks global, I. DOI: 10.1107/S1600536809009076/hk2642sup1.cif

Structure factors: contains datablocks I. DOI: 10.1107/S1600536809009076/hk2642Isup2.hkl

Additional supplementary materials:  crystallographic information; 3D view; checkCIF report

## Figures and Tables

**Table 1:** Hydrogen-bond geometry (Å, °)

*D*—H⋯*A*	*D*—H	H⋯*A*	*D*⋯*A*	*D*—H⋯*A*
C10—H10*B*⋯O4^i^	0.97	2.58	3.336 (7)	135
C13—H13*B*⋯O9^ii^	0.96	2.41	3.211 (6)	141
C21—H21*A*⋯O5^iii^	0.96	2.48	3.243 (5)	136
C24—H24*A*⋯O9^iv^	0.97	2.59	3.276 (6)	128

Symmetry codes: (i) 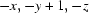; (ii) 
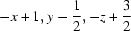
; (iii) 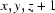; (iv) 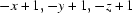.

**Fig. 1.**
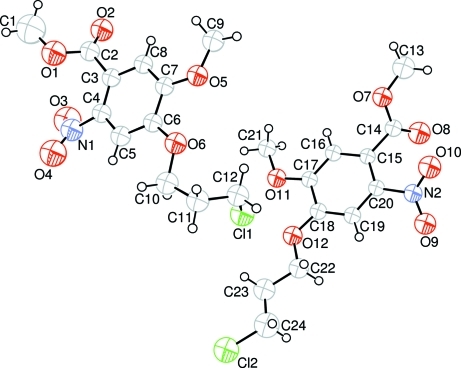
The molecular structure of the title molecule, with the atom-numbering scheme.

**Fig. 2.**
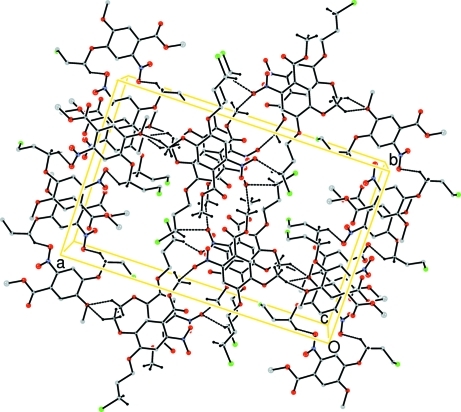
A partial packing diagram of the title compound. Hydrogen bonds are shown as dashed lines. H atoms not involved in hydrogen bonding are omitted.

## supplementary crystallographic information

### Comment

As part of our ongoing studies on quinazoline derivatives (Knesl *et al.*, 2006), we report herein the crystal structure of the title compound.

The asymmetric unit of the title compound contains two crystallographically independent molecules (Fig. 1), in which the bond lengths (Allen *et al.*, 1987) and angles are within normal ranges. Rings A (C3-C8) and A' (C15-C20) are, of course, planar and they are oriented at a dihedral angle of A/A' = 9.12 (3)°.

In the crystal structure, weak intermolecular C-H···O hydrogen bonds (Table 1) link the molecules into a three dimensional network (Fig. 2), in which they may be effective in the stabilization of the structure.

### Experimental

For the preparation of the title compound, a solution of methyl 4-(3-chloro- propoxy)-3-methoxybenzoate (19 mmol) in acetic acid (20 ml) was added dropwise to nitric acid (98%, 4.5 ml) at 273-278 K. The mixture was stirred for 1 h at room temperature, and then for 2 h at 323 K. After the reaction was completed, the reaction mixture was poured into ice/water (130 ml), and then extracted with trichloromethane (20 ml). The combined organic phases were collected, washed with saturated sodium bicarbonate (20 ml), brine (20 ml), dried (Na_2_SO_4_) and decolorized (charcoal). Trichloromethane was then removed under reduced pressure to give a yellow oil, which was crystallized from ethyl acetate/petroleum ether to afford the product as light yellow crystals (m.p. 337 K). Crystals suitable for X-ray analysis were obtained by slow evaporation of a methanol solution.

### Refinement

H atoms were positioned geometrically, with C-H = 0.93, 0.97 and 0.96 Å for aromatic, methylene and methyl H, respectively, and constrained to ride on their parent atoms, with U_iso_(H) = xU_eq_(C), where x = 1.5 for methyl H and x = 1.2 for all other H atoms.

### Crystal data

**Table:**

C_12_H_14_ClNO_6_	*F*(000) = 1264
*M**_r_* = 303.69	*D*_x_ = 1.441 Mg m^−^^3^
Monoclinic, *P*2_1_/*c*	Melting point: 337 K
Hall symbol: -P 2ybc	Mo *K*α radiation, λ = 0.71073 Å
*a* = 23.150 (5) Å	Cell parameters from 25 reflections
*b* = 15.013 (3) Å	θ = 10–13°
*c* = 8.0700 (16) Å	µ = 0.30 mm^−^^1^
β = 93.42 (3)°	*T* = 294 K
*V* = 2799.7 (10) Å^3^	Needle, yellow
*Z* = 8	0.30 × 0.20 × 0.20 mm

### Data collection

**Table:**

Enraf–Nonius CAD-4 diffractometer	2874 reflections with *I* > 2σ(*I*)
Radiation source: fine-focus sealed tube	*R*_int_ = 0.038
graphite	θ_max_ = 25.3°, θ_min_ = 1.6°
ω/2θ scans	*h* = 0→27
Absorption correction: ψ scan (North *et al.*, 1968)	*k* = 0→18
*T*_min_ = 0.916, *T*_max_ = 0.943	*l* = −9→9
5208 measured reflections	3 standard reflections every 120 min
5096 independent reflections	intensity decay: 1%

### Refinement

**Table:**

Refinement on *F*^2^	Secondary atom site location: difference Fourier map
Least-squares matrix: full	Hydrogen site location: inferred from neighbouring sites
*R*[*F*^2^ > 2σ(*F*^2^)] = 0.065	H-atom parameters constrained
*wR*(*F*^2^) = 0.162	*w* = 1/[σ^2^(*F*_o_^2^) + (0.0567*P*)^2^ + 1.915*P*] where *P* = (*F*_o_^2^ + 2*F*_c_^2^)/3
*S* = 1.03	(Δ/σ)_max_ < 0.001
5096 reflections	Δρ_max_ = 0.40 e Å^−^^3^
362 parameters	Δρ_min_ = −0.30 e Å^−^^3^
0 restraints	Extinction correction: *SHELXL97* (Sheldrick, 2008), Fc^*^=kFc[1+0.001xFc^2^λ^3^/sin(2θ)]^-1/4^
Primary atom site location: structure-invariant direct methods	Extinction coefficient: 0.0049 (6)

### Special details

**Table:**

Geometry. All esds (except the esd in the dihedral angle between two l.s. planes) are estimated using the full covariance matrix. The cell esds are taken into account individually in the estimation of esds in distances, angles and torsion angles; correlations between esds in cell parameters are only used when they are defined by crystal symmetry. An approximate (isotropic) treatment of cell esds is used for estimating esds involving l.s. planes.
Refinement. Refinement of F^2^ against ALL reflections. The weighted R-factor wR and goodness of fit S are based on F^2^, conventional R-factors R are based on F, with F set to zero for negative F^2^. The threshold expression of F^2^ > 2sigma(F^2^) is used only for calculating R-factors(gt) etc. and is not relevant to the choice of reflections for refinement. R-factors based on F^2^ are statistically about twice as large as those based on F, and R- factors based on ALL data will be even larger.

### Fractional atomic coordinates and isotropic or equivalent isotropic displacement parameters (Å^2^)

**Table:**

	*x*	*y*	*z*	*U*_iso_*/*U*_eq_	
Cl1	0.26534 (6)	0.48992 (8)	0.29390 (17)	0.0758 (4)	
Cl2	0.30461 (6)	0.79461 (8)	0.6560 (2)	0.0888 (5)	
O1	−0.10537 (13)	0.2705 (2)	0.1972 (5)	0.0846 (11)	
O2	−0.05735 (14)	0.1596 (2)	0.3282 (4)	0.0714 (9)	
O3	−0.06317 (19)	0.3963 (3)	0.4296 (5)	0.1081 (15)	
O4	−0.05061 (16)	0.5014 (2)	0.2559 (6)	0.0970 (13)	
O5	0.13459 (12)	0.23218 (18)	0.0445 (4)	0.0559 (8)	
O6	0.14060 (12)	0.40304 (18)	0.0445 (4)	0.0593 (8)	
O7	0.40610 (12)	0.06586 (17)	0.5910 (4)	0.0558 (8)	
O8	0.48185 (14)	0.1024 (2)	0.7607 (4)	0.0740 (10)	
O9	0.53876 (12)	0.3392 (2)	0.6019 (4)	0.0637 (9)	
O10	0.51561 (13)	0.2122 (2)	0.4944 (4)	0.0694 (9)	
O11	0.28231 (11)	0.33078 (17)	0.7787 (4)	0.0528 (7)	
O12	0.34201 (12)	0.46634 (17)	0.7009 (4)	0.0538 (8)	
N1	−0.04080 (17)	0.4270 (3)	0.3109 (6)	0.0687 (11)	
N2	0.50447 (14)	0.2793 (2)	0.5715 (4)	0.0466 (8)	
C1	−0.1592 (2)	0.2271 (5)	0.2275 (9)	0.124 (3)	
H1A	−0.1907	0.2607	0.1761	0.185*	
H1B	−0.1635	0.2237	0.3449	0.185*	
H1C	−0.1593	0.1681	0.1816	0.185*	
C2	−0.05727 (18)	0.2299 (3)	0.2610 (6)	0.0554 (11)	
C3	−0.00469 (17)	0.2813 (3)	0.2209 (5)	0.0477 (10)	
C4	0.00118 (18)	0.3722 (3)	0.2282 (5)	0.0495 (10)	
C5	0.04788 (18)	0.4163 (3)	0.1691 (5)	0.0542 (11)	
H5A	0.0498	0.4781	0.1724	0.065*	
C6	0.09190 (17)	0.3675 (3)	0.1046 (5)	0.0474 (10)	
C7	0.08873 (17)	0.2742 (3)	0.1040 (5)	0.0463 (10)	
C8	0.04040 (17)	0.2321 (3)	0.1598 (5)	0.0476 (10)	
H8A	0.0380	0.1702	0.1564	0.057*	
C9	0.1376 (2)	0.1372 (3)	0.0606 (6)	0.0626 (12)	
H9A	0.1721	0.1159	0.0133	0.094*	
H9B	0.1043	0.1109	0.0033	0.094*	
H9C	0.1384	0.1213	0.1759	0.094*	
C10	0.1491 (2)	0.4975 (3)	0.0631 (7)	0.0645 (13)	
H10A	0.1494	0.5140	0.1794	0.077*	
H10B	0.1182	0.5297	0.0031	0.077*	
C11	0.2060 (2)	0.5190 (3)	−0.0057 (6)	0.0638 (13)	
H11A	0.2128	0.5825	0.0057	0.077*	
H11B	0.2037	0.5053	−0.1234	0.077*	
C12	0.25688 (19)	0.4705 (3)	0.0750 (6)	0.0612 (12)	
H12A	0.2918	0.4893	0.0241	0.073*	
H12B	0.2521	0.4071	0.0553	0.073*	
C13	0.4229 (2)	−0.0264 (3)	0.6021 (7)	0.0748 (15)	
H13A	0.3962	−0.0617	0.5344	0.112*	
H13B	0.4226	−0.0458	0.7154	0.112*	
H13C	0.4611	−0.0332	0.5638	0.112*	
C14	0.43940 (17)	0.1222 (3)	0.6788 (5)	0.0433 (10)	
C15	0.41457 (15)	0.2145 (2)	0.6687 (4)	0.0367 (9)	
C16	0.35887 (16)	0.2280 (2)	0.7183 (5)	0.0409 (9)	
H16A	0.3365	0.1790	0.7442	0.049*	
C17	0.33587 (16)	0.3122 (2)	0.7300 (5)	0.0397 (9)	
C18	0.36847 (16)	0.3871 (2)	0.6871 (5)	0.0415 (9)	
C19	0.42370 (16)	0.3744 (2)	0.6376 (5)	0.0418 (9)	
H19A	0.4460	0.4231	0.6098	0.050*	
C20	0.44601 (15)	0.2890 (2)	0.6293 (4)	0.0378 (9)	
C21	0.24745 (17)	0.2586 (3)	0.8302 (5)	0.0521 (11)	
H21A	0.2108	0.2811	0.8613	0.078*	
H21B	0.2667	0.2292	0.9235	0.078*	
H21C	0.2414	0.2171	0.7404	0.078*	
C22	0.3705 (2)	0.5435 (3)	0.6460 (6)	0.0567 (11)	
H22A	0.3781	0.5384	0.5295	0.068*	
H22B	0.4070	0.5523	0.7096	0.068*	
C23	0.3290 (2)	0.6207 (3)	0.6738 (7)	0.0680 (14)	
H23A	0.2931	0.6108	0.6080	0.082*	
H23B	0.3201	0.6222	0.7897	0.082*	
C24	0.3531 (2)	0.7046 (3)	0.6292 (8)	0.0895 (18)	
H24A	0.3629	0.7024	0.5141	0.107*	
H24B	0.3885	0.7149	0.6969	0.107*	

### Atomic displacement parameters (Å^2^)

**Table:**

	*U*^11^	*U*^22^	*U*^33^	*U*^12^	*U*^13^	*U*^23^
Cl1	0.0858 (9)	0.0698 (8)	0.0709 (8)	−0.0107 (7)	−0.0018 (7)	−0.0109 (7)
Cl2	0.0811 (9)	0.0518 (7)	0.1376 (13)	0.0225 (6)	0.0417 (9)	0.0179 (8)
O1	0.049 (2)	0.095 (3)	0.111 (3)	−0.0011 (18)	0.0157 (19)	0.046 (2)
O2	0.071 (2)	0.066 (2)	0.079 (2)	−0.0029 (17)	0.0184 (18)	0.0170 (19)
O3	0.124 (3)	0.119 (3)	0.087 (3)	0.048 (3)	0.053 (3)	0.017 (3)
O4	0.088 (3)	0.059 (2)	0.148 (4)	0.018 (2)	0.037 (2)	0.006 (2)
O5	0.0529 (18)	0.0488 (17)	0.068 (2)	−0.0020 (14)	0.0179 (15)	−0.0026 (15)
O6	0.0515 (18)	0.0475 (17)	0.080 (2)	−0.0126 (14)	0.0128 (16)	−0.0039 (15)
O7	0.0585 (18)	0.0382 (15)	0.069 (2)	0.0053 (14)	−0.0072 (15)	−0.0044 (15)
O8	0.064 (2)	0.064 (2)	0.089 (2)	0.0219 (17)	−0.0301 (19)	−0.0083 (18)
O9	0.0436 (17)	0.071 (2)	0.078 (2)	−0.0114 (16)	0.0142 (15)	−0.0192 (17)
O10	0.062 (2)	0.060 (2)	0.089 (2)	0.0059 (16)	0.0290 (18)	−0.0250 (18)
O11	0.0425 (16)	0.0458 (16)	0.072 (2)	0.0063 (13)	0.0190 (14)	−0.0015 (15)
O12	0.0547 (18)	0.0370 (15)	0.072 (2)	0.0082 (13)	0.0231 (15)	0.0010 (14)
N1	0.062 (3)	0.070 (3)	0.075 (3)	0.008 (2)	0.011 (2)	0.000 (2)
N2	0.041 (2)	0.051 (2)	0.049 (2)	0.0044 (17)	0.0083 (16)	−0.0039 (18)
C1	0.048 (3)	0.155 (6)	0.171 (7)	−0.014 (4)	0.023 (4)	0.067 (5)
C2	0.048 (3)	0.068 (3)	0.052 (3)	0.001 (2)	0.014 (2)	0.001 (2)
C3	0.044 (2)	0.056 (3)	0.043 (2)	0.004 (2)	0.0049 (19)	0.001 (2)
C4	0.046 (2)	0.055 (3)	0.048 (3)	0.005 (2)	0.005 (2)	−0.008 (2)
C5	0.052 (3)	0.045 (2)	0.065 (3)	−0.001 (2)	−0.003 (2)	−0.003 (2)
C6	0.043 (2)	0.051 (3)	0.048 (3)	−0.005 (2)	0.000 (2)	−0.001 (2)
C7	0.048 (3)	0.046 (2)	0.044 (2)	−0.002 (2)	0.000 (2)	−0.006 (2)
C8	0.050 (3)	0.044 (2)	0.049 (3)	−0.005 (2)	0.006 (2)	0.001 (2)
C9	0.063 (3)	0.049 (3)	0.078 (3)	−0.001 (2)	0.017 (2)	−0.006 (2)
C10	0.060 (3)	0.042 (2)	0.092 (4)	−0.007 (2)	0.002 (3)	0.003 (2)
C11	0.073 (3)	0.047 (3)	0.071 (3)	−0.014 (2)	0.004 (3)	0.005 (2)
C12	0.058 (3)	0.059 (3)	0.067 (3)	−0.009 (2)	0.013 (2)	−0.007 (2)
C13	0.082 (4)	0.036 (2)	0.106 (4)	0.005 (2)	0.001 (3)	0.000 (3)
C14	0.044 (2)	0.046 (2)	0.041 (2)	0.004 (2)	0.0054 (19)	−0.0019 (19)
C15	0.038 (2)	0.039 (2)	0.033 (2)	0.0017 (17)	0.0013 (17)	−0.0016 (17)
C16	0.041 (2)	0.036 (2)	0.046 (2)	−0.0013 (17)	0.0048 (18)	0.0002 (18)
C17	0.036 (2)	0.044 (2)	0.040 (2)	0.0053 (18)	0.0084 (17)	−0.0025 (18)
C18	0.046 (2)	0.039 (2)	0.041 (2)	0.0089 (19)	0.0060 (18)	−0.0043 (18)
C19	0.049 (2)	0.039 (2)	0.039 (2)	−0.0025 (18)	0.0081 (18)	−0.0030 (18)
C20	0.035 (2)	0.044 (2)	0.035 (2)	0.0052 (17)	0.0059 (16)	−0.0034 (17)
C21	0.046 (2)	0.058 (3)	0.053 (3)	−0.004 (2)	0.013 (2)	−0.004 (2)
C22	0.068 (3)	0.040 (2)	0.063 (3)	0.007 (2)	0.016 (2)	0.001 (2)
C23	0.074 (3)	0.046 (3)	0.087 (4)	0.006 (2)	0.030 (3)	−0.001 (3)
C24	0.080 (4)	0.061 (3)	0.131 (5)	0.013 (3)	0.036 (4)	−0.002 (3)

### Geometric parameters (Å, °)

**Table:**

Cl1—C12	1.789 (5)	C9—H9A	0.9600
Cl2—C24	1.778 (5)	C9—H9B	0.9600
O1—C2	1.345 (5)	C9—H9C	0.9600
O1—C1	1.439 (5)	C10—C11	1.496 (6)
O2—C2	1.187 (5)	C10—H10A	0.9700
O3—N1	1.208 (5)	C10—H10B	0.9700
O4—N1	1.218 (5)	C11—C12	1.500 (6)
O5—C7	1.348 (5)	C11—H11A	0.9700
O5—C9	1.433 (5)	C11—H11B	0.9700
O6—C6	1.363 (4)	C12—H12A	0.9700
O6—C10	1.438 (5)	C12—H12B	0.9700
O7—C14	1.322 (5)	C13—H13A	0.9600
O7—C13	1.440 (5)	C13—H13B	0.9600
O8—C14	1.189 (4)	C13—H13C	0.9600
O9—N2	1.215 (4)	C14—C15	1.501 (5)
O10—N2	1.220 (4)	C15—C20	1.382 (5)
O11—C17	1.352 (4)	C15—C16	1.388 (5)
O11—C21	1.427 (4)	C16—C17	1.377 (5)
O12—C18	1.346 (4)	C16—H16A	0.9300
O12—C22	1.417 (5)	C17—C18	1.409 (5)
N1—C4	1.464 (5)	C18—C19	1.375 (5)
N2—C20	1.465 (5)	C19—C20	1.385 (5)
C1—H1A	0.9600	C19—H19A	0.9300
C1—H1B	0.9600	C21—H21A	0.9600
C1—H1C	0.9600	C21—H21B	0.9600
C2—C3	1.493 (6)	C21—H21C	0.9600
C3—C4	1.372 (5)	C22—C23	1.530 (5)
C3—C8	1.393 (5)	C22—H22A	0.9700
C4—C5	1.377 (6)	C22—H22B	0.9700
C5—C6	1.381 (5)	C23—C24	1.432 (6)
C5—H5A	0.9300	C23—H23A	0.9700
C6—C7	1.402 (5)	C23—H23B	0.9700
C7—C8	1.384 (5)	C24—H24A	0.9700
C8—H8A	0.9300	C24—H24B	0.9700
			
C2—O1—C1	115.8 (4)	C11—C12—Cl1	112.7 (3)
C7—O5—C9	118.0 (3)	C11—C12—H12A	109.0
C6—O6—C10	117.4 (3)	Cl1—C12—H12A	109.0
C14—O7—C13	115.8 (3)	C11—C12—H12B	109.0
C17—O11—C21	118.2 (3)	Cl1—C12—H12B	109.0
C18—O12—C22	118.3 (3)	H12A—C12—H12B	107.8
O3—N1—O4	124.0 (4)	O7—C13—H13A	109.5
O3—N1—C4	118.3 (4)	O7—C13—H13B	109.5
O4—N1—C4	117.6 (4)	H13A—C13—H13B	109.5
O9—N2—O10	124.0 (3)	O7—C13—H13C	109.5
O9—N2—C20	117.9 (3)	H13A—C13—H13C	109.5
O10—N2—C20	118.2 (3)	H13B—C13—H13C	109.5
O1—C1—H1A	109.5	O8—C14—O7	125.0 (4)
O1—C1—H1B	109.5	O8—C14—C15	124.2 (4)
H1A—C1—H1B	109.5	O7—C14—C15	110.7 (3)
O1—C1—H1C	109.5	C20—C15—C16	117.3 (3)
H1A—C1—H1C	109.5	C20—C15—C14	123.7 (3)
H1B—C1—H1C	109.5	C16—C15—C14	118.6 (3)
O2—C2—O1	123.7 (4)	C17—C16—C15	121.6 (3)
O2—C2—C3	125.6 (4)	C17—C16—H16A	119.2
O1—C2—C3	110.5 (4)	C15—C16—H16A	119.2
C4—C3—C8	118.0 (4)	O11—C17—C16	125.1 (3)
C4—C3—C2	125.8 (4)	O11—C17—C18	114.9 (3)
C8—C3—C2	116.1 (4)	C16—C17—C18	120.0 (3)
C3—C4—C5	122.7 (4)	O12—C18—C19	125.6 (4)
C3—C4—N1	120.8 (4)	O12—C18—C17	115.6 (3)
C5—C4—N1	116.4 (4)	C19—C18—C17	118.8 (3)
C4—C5—C6	119.2 (4)	C18—C19—C20	119.8 (3)
C4—C5—H5A	120.4	C18—C19—H19A	120.1
C6—C5—H5A	120.4	C20—C19—H19A	120.1
O6—C6—C5	124.9 (4)	C15—C20—C19	122.3 (3)
O6—C6—C7	115.7 (4)	C15—C20—N2	120.1 (3)
C5—C6—C7	119.4 (4)	C19—C20—N2	117.5 (3)
O5—C7—C8	124.8 (4)	O11—C21—H21A	109.5
O5—C7—C6	115.3 (3)	O11—C21—H21B	109.5
C8—C7—C6	119.9 (4)	H21A—C21—H21B	109.5
C7—C8—C3	120.6 (4)	O11—C21—H21C	109.5
C7—C8—H8A	119.7	H21A—C21—H21C	109.5
C3—C8—H8A	119.7	H21B—C21—H21C	109.5
O5—C9—H9A	109.5	O12—C22—C23	105.3 (3)
O5—C9—H9B	109.5	O12—C22—H22A	110.7
H9A—C9—H9B	109.5	C23—C22—H22A	110.7
O5—C9—H9C	109.5	O12—C22—H22B	110.7
H9A—C9—H9C	109.5	C23—C22—H22B	110.7
H9B—C9—H9C	109.5	H22A—C22—H22B	108.8
O6—C10—C11	107.0 (4)	C24—C23—C22	111.8 (4)
O6—C10—H10A	110.3	C24—C23—H23A	109.2
C11—C10—H10A	110.3	C22—C23—H23A	109.2
O6—C10—H10B	110.3	C24—C23—H23B	109.2
C11—C10—H10B	110.3	C22—C23—H23B	109.2
H10A—C10—H10B	108.6	H23A—C23—H23B	107.9
C10—C11—C12	114.8 (4)	C23—C24—Cl2	112.4 (4)
C10—C11—H11A	108.6	C23—C24—H24A	109.1
C12—C11—H11A	108.6	Cl2—C24—H24A	109.1
C10—C11—H11B	108.6	C23—C24—H24B	109.1
C12—C11—H11B	108.6	Cl2—C24—H24B	109.1
H11A—C11—H11B	107.5	H24A—C24—H24B	107.9
			
C1—O1—C2—O2	5.3 (7)	C13—O7—C14—O8	1.8 (6)
C1—O1—C2—C3	179.9 (5)	C13—O7—C14—C15	−175.1 (3)
O2—C2—C3—C4	−143.1 (5)	O8—C14—C15—C20	53.4 (6)
O1—C2—C3—C4	42.5 (6)	O7—C14—C15—C20	−129.7 (4)
O2—C2—C3—C8	41.7 (6)	O8—C14—C15—C16	−119.6 (5)
O1—C2—C3—C8	−132.8 (4)	O7—C14—C15—C16	57.4 (5)
C8—C3—C4—C5	3.9 (6)	C20—C15—C16—C17	−0.8 (5)
C2—C3—C4—C5	−171.3 (4)	C14—C15—C16—C17	172.6 (4)
C8—C3—C4—N1	−171.9 (4)	C21—O11—C17—C16	3.3 (6)
C2—C3—C4—N1	12.9 (7)	C21—O11—C17—C18	−177.7 (3)
O3—N1—C4—C3	33.0 (7)	C15—C16—C17—O11	−179.4 (3)
O4—N1—C4—C3	−147.6 (4)	C15—C16—C17—C18	1.6 (6)
O3—N1—C4—C5	−143.1 (5)	C22—O12—C18—C19	6.2 (6)
O4—N1—C4—C5	36.3 (6)	C22—O12—C18—C17	−174.6 (4)
C3—C4—C5—C6	−2.3 (7)	O11—C17—C18—O12	0.2 (5)
N1—C4—C5—C6	173.7 (4)	C16—C17—C18—O12	179.3 (3)
C10—O6—C6—C5	5.8 (6)	O11—C17—C18—C19	179.4 (3)
C10—O6—C6—C7	−172.0 (4)	C16—C17—C18—C19	−1.5 (6)
C4—C5—C6—O6	−179.1 (4)	O12—C18—C19—C20	179.7 (4)
C4—C5—C6—C7	−1.4 (6)	C17—C18—C19—C20	0.6 (6)
C9—O5—C7—C8	−9.0 (6)	C16—C15—C20—C19	−0.2 (5)
C9—O5—C7—C6	172.0 (4)	C14—C15—C20—C19	−173.2 (4)
O6—C6—C7—O5	0.3 (5)	C16—C15—C20—N2	−178.3 (3)
C5—C6—C7—O5	−177.6 (4)	C14—C15—C20—N2	8.7 (6)
O6—C6—C7—C8	−178.8 (3)	C18—C19—C20—C15	0.3 (6)
C5—C6—C7—C8	3.3 (6)	C18—C19—C20—N2	178.4 (3)
O5—C7—C8—C3	179.3 (4)	O9—N2—C20—C15	−150.4 (4)
C6—C7—C8—C3	−1.7 (6)	O10—N2—C20—C15	30.4 (5)
C4—C3—C8—C7	−1.8 (6)	O9—N2—C20—C19	31.4 (5)
C2—C3—C8—C7	173.8 (4)	O10—N2—C20—C19	−147.8 (4)
C6—O6—C10—C11	177.8 (4)	C18—O12—C22—C23	178.5 (4)
O6—C10—C11—C12	−58.2 (5)	O12—C22—C23—C24	177.8 (5)
C10—C11—C12—Cl1	−57.8 (5)	C22—C23—C24—Cl2	178.6 (4)

### Hydrogen-bond geometry (Å, °)

**Table:**

*D*—H···*A*	*D*—H	H···*A*	*D*···*A*	*D*—H···*A*
C10—H10B···O4^i^	0.97	2.58	3.336 (7)	135
C13—H13B···O9^ii^	0.96	2.41	3.211 (6)	141
C21—H21A···O5^iii^	0.96	2.48	3.243 (5)	136
C24—H24A···O9^iv^	0.97	2.59	3.276 (6)	128

Symmetry codes: (i) −*x*, −*y*+1, −*z*; (ii) −*x*+1, *y*−1/2, −*z*+3/2; (iii) *x*, *y*, *z*+1; (iv) −*x*+1, −*y*+1, −*z*+1.

## References

[bb1] Allen, F. H., Kennard, O., Watson, D. G., Brammer, L., Orpen, A. G. & Taylor, R. (1987). *J. Chem. Soc. Perkin Trans. 2*, pp. S1–19.

[bb2] Enraf–Nonius (1989). *CAD-4 Software* Enraf–Nonius, Delft. The Netherlands.

[bb3] Farrugia, L. J. (1997). *J. Appl. Cryst.* **30**, 565.

[bb4] Harms, K. & Wocadlo, S. (1995). *XCAD4* University of Marburg, Germany.

[bb5] Knesl, P., Roeseling, D. & Jordis, U. (2006). *Molecules*, **11**, 286–297. 10.3390/11040286 PMC6148630 17962760

[bb6] North, A. C. T., Phillips, D. C. & Mathews, F. S. (1968). *Acta Cryst.* A**24**, 351–359.

[bb7] Sheldrick, G. M. (2008). *Acta Cryst.* A**64**, 112–122. 10.1107/S0108767307043930 18156677

